# The vicious cycle: a history of obesity and COVID-19

**DOI:** 10.1186/s12872-021-02134-y

**Published:** 2021-07-06

**Authors:** Jacek Bil, Olga Możeńska

**Affiliations:** 1 Department of Invasive Cardiology, Centre of Postgraduate Medical Education, Woloska Street 137, 02-507 Warsaw, Poland; 2 Department of Internal Medicine, Hypertension and Angiology, Independent Public Central Clinical Hospital, Medical University of Warsaw, Warsaw, Poland

**Keywords:** Coronavirus disease-related cardiometabolic syndrome, Statins, SARS-CoV-2, High-processed food, ACE2, GLP-1

## Abstract

Recently, we face a surge in the fast-forward Coronavirus Disease 2019 (COVID-19) pandemic with nearly 170 million confirmed cases and almost 3.5 million confirmed deaths at the end of May 2021. Obesity, also known as the pandemic of the 21st century, has been evolving as an adverse prognostic marker. Obesity is associated with a higher risk of being SARS-CoV-2-positive (46%), as well as hospitalization (113%) and death (48%) due to COVID-19. It is especially true for subjects with morbid obesity. Also, observational studies suggest that in the case of COVID-19, no favorable “obesity paradox” is observed. Therefore, it is postulated to introduce a new entity, i.e., coronavirus disease-related cardiometabolic syndrome (CIRCS). In theory, it applies to all stages of COVID-19, i.e., prevention, acute proceedings (from COVID-19 diagnosis to resolution or three months), and long-term outcomes. Consequently, lifestyle changes, glycemic control, and regulation of the renin-angiotensin-aldosterone pathway have crucial implications for preventing and managing subjects with COVID-19. Finally, it is crucial to use cardioprotective drugs such as angiotensin-converting enzyme inhibitors/angiotensin II receptor blockers and statins. Nevertheless, there is the need to conduct prospective studies and registries better to evaluate the issue of obesity in COVID-19 patients.

## Background

Recently, we face a surge in the fast-forward Coronavirus Disease 2019 (COVID-19) pandemic with nearly 170 million confirmed cases and almost 3.5 million confirmed deaths at the end of May 2021 [1]. Recently, in BMC Cardiovascular Disorders, several interesting papers have been published focusing on risk factors of severe prognosis and COVID-19 complications.

Li et al. analyzed data of 100 subjects with a severe type of COVID-19 [2]. The multivariable analysis found that male sex (HR 5.09, 95% CI 1.19–22.17) and hypertension (HR 9.88, 95% CI 2.52–28.70) were the risk factors of cardiac injury. The cardiac injury was observed in 25% of cases, and the mortality was 4.0%. Unfortunately, the authors did not assess the role and impact of obesity in the COVID-19 course. Sardu et al. evaluated the effect of ABO blood groups on outcomes in patients with hypertension and COVID-19 [3]. Blood group other than O was associated with a higher risk of cardiac injury (HR 2.57, 95% CI 1.21–5.49) and death (HR 3.71, 95% CI 1.22–11.24). And finally, Silverio et al., in a large meta-analysis (45 studies, over 18,000 subjects), showed that only diabetes was related to in-hospital mortality in subjects with COVID-19 [4].

However, during COVID-19, another critical factor has emerged, i.e., obesity, defined as body mass index over 30.0 kg/m^2^. Obesity, also known as the pandemic of the 21st century, has evolved as a prognostic marker of worse outcomes. Unfortunately, home isolation, home office, and sedentarism during the COVID-19 pandemic intensify the problem of overweight and obesity—the perfect vicious cycle [5]. Indeed, in large-scale studies, even over 30% of respondents confirmed weight gain during the lockdown (19.5 − 31.5%). Moreover, overweight or obese respondents were more likely to report weight gain during the pandemic than respondents with normal body weight [6].

## Main text

### Obesity as a negative prognostic marker

Popkin et al., in a recent huge meta-analysis (75 studies, 399,461 patients from Asia, Europe, North America, and South America), proved that subjects with obesity were at a higher risk of being SARS-CoV-2-positive (OR 1.46, 95% CI 1.30–1.65) as well as had a higher risk of complications in the COVID-19 course (hospitalization—OR 2.13, 95% CI 1.74–2.60; intensive care unit admission—OR 1.74, 95% CI 1.46–2.08; and in-hospital death—OR 1.48, 95% CI 1.22–1.80) [7]. The most recent studies focusing on COVID-19 course and obesity are provided in Table 1 [8–12]. In some of these studies, obesity was defined as BMI > 28 kg/m^2^ (especially in the Asian population) [8], and in some, the significant impact of COVID-19 was observed only in subjects with morbid obesity with BMI 40–45 kg/m^2^ [10, 12].

**Table 1:** Obesity as a risk factor for the worse COVID-19 course

Study	Patients	Hospitalization OR (95% CI)	ICU admission OR (95% CI)	In-hospital death OR (95% CI)	Severe course OR (95% CI)
Popkin [7]	399,461	2.13 (1.74–2.60)	1.74 (1.46–2.08)	1.48 (1.22–1.80)	–
Cai [8]	383	–	–	–	3.40 (1.40–2.86)
Simonnet [9]	124	–	7.36 (1.63–33.14)^a^	–	–
Petrilli [10]^b^	5,279	–	–	1.45 (0.99–2.13)	1.71 (1.10–2.7)
Yates [11]	54,254	–	3.91 (3.13–4.88) 5.03 (3.94 -6.63)^a^	1.93 (1.49–2.51)	–
Kompaniyets [12]^b^	148,494	1.33 (1.30–1.37)	1.16 (1.11–1.20) 2.08 (1.89–2.29)^a^	1.61 (1.47 − 1.76)	–

*OR* odds ratio,* CI* confidence interva,* ICU* intensive care unit

^a^Requiring invasive mechanical ventilation

^b^BMI > 40 kg/m^2^

Interestingly, this was confirmed in a meta-analysis of 76 studies with 17,860,001 subjects. This meta-analysis showed that the worse prognosis of COVID-19 was observed in subjects over 75 years of age, males, and severe obesity (OR 2.57, 95% CI 1.31–5.05) [13]. Also, data from HOPE COVID-19 Registry did not support the presence of, known from other disease entities, the potentially favorable “obesity paradox” in subjects with COVID-19 [14].

### Coronavirus disease-related cardiometabolic syndrome

**Fig. 1:**
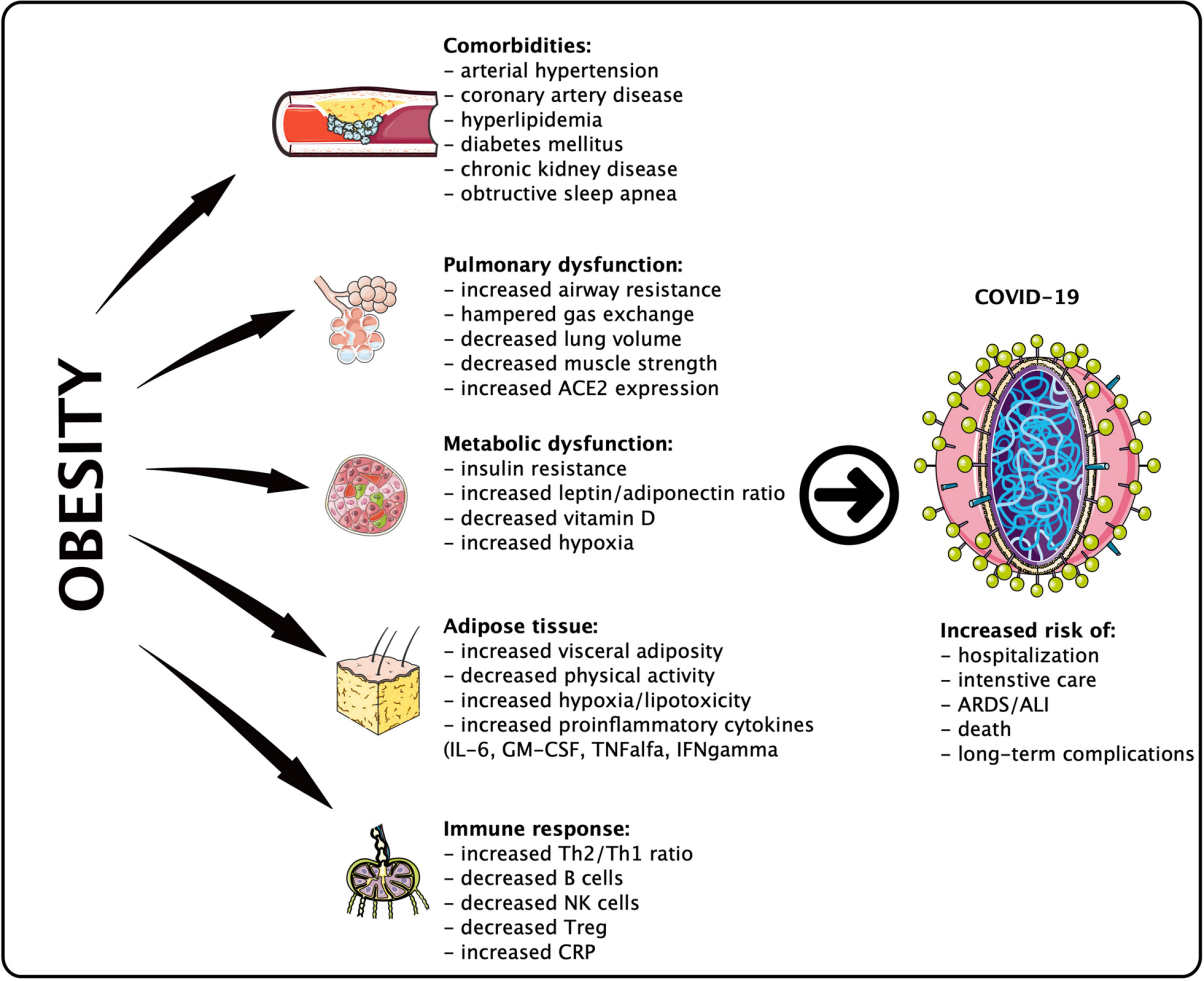
The influence of obesity on the severe course of COVID-19. *ACE2* angiotensin converting enzyme 2, *ALI* acute lung injury, *ARDS* acute respiratory distress syndrome, *CRP* C-reactive protein, *GM-CSF* granulocyte-macrophage colony stimulating factor, *IFNgamma* interferon gamma, *IL-6* interleukin 6, *TNFalfa* tumor necrosis factor alfa

However, the question persists why obese subjects are at risk for severe COVID-19 course? Obesity per se is a metabolic entity characterized by systemic metabolism changes, such as insulin resistance, increased serum glucose, a high leptin/adiponectin ratio, and a persistent low-grade inflammatory state [15]. Obesity is a key player in classical cardiometabolic syndrome. Moreover, vascular and lung function alterations, impaired immune response, and viral-bacterial interactions may play a significant role (Fig. 1) [16].

Sarver et al. proved diet- and gender-dependent changes in angiotensin-converting enzyme 2 (ACE2) expression in the trachea and lungs [15]. ACE2 expression was increased in the lungs and trachea of diet-induced obese male mice comparing with lean subjects. Also, in diet-induced obese mice, males characterized more pronounced ACE2 expression in the trachea than females. And ACE2 upregulation may predispose to SARS-CoV-2 infection. Consequently, fatty tissue in subjects with obesity may behave as a milieu for more intense SARS-CoV-2 replication. The large volume of adipose tissue (especially in males) may promote accelerated viral shedding and exaggerated immune response leading to severe complications [18, 19].

Therefore, it is postulated to introduce a new entity, i.e., coronavirus disease-related cardiometabolic syndrome (CIRCS) [20]. It applies to all stages of COVID-19, including its prevention, acute proceedings (from COVID-19 diagnosis to resolution or three months), and long-term outcomes. Components of acute CIRCS include abnormal adiposity, cardiovascular diseases, acute kidney injury, severe acute respiratory syndrome, high insensible water losses, and hypernatremia, encephalopathy, hypercoagulable state, and thromboembolism as well as metabolic disturbances (hypercytokinemia, inflammatory state, severe insulin resistance, hyperglycemia, hyperphosphatemia, and hypocalcemia). Such subjects are at high risk of the unfavorable COVID-19 course, and therefore, we should aggressively manage crucial metabolic risk factors of cardiovascular disorders in COVID-19 subjects (especially those with obesity) [20].

### Pharmacotherapy options to improve outcome in obese COVID-19 subjects

One of the crucial elements is the renin-angiotensin-aldosterone pathway. Angiotensin II receptor type 1 (AT-1) blockade by angiotensin II receptor blockers (ARB) or inhibition of angiotensin II formation by ACE inhibitors, frequently administered in obese subjects with arterial hypertension and diabetes mellitus, may theoretically predispose to an increase in transmembrane ACE2 levels with a simultaneous decrease in soluble ACE2 levels. Nevertheless, there is a broad agreement amid professional medical societies in Europe and America to carry on with renin-angiotensin-aldosterone pathway inhibitors in subjects currently taking these medications. Zhang et al. proved that subjects treated with ACE inhibitors/ARB characterized a 63% lower risk of COVID-19 death than subjects who did not receive ACE inhibitors/ARB [21].

Statins are emerging as another group of drugs that could reduce the risk of unfavorable outcomes in subjects with COVID-19. Kow et al., in a meta-analysis, revealed a significantly decreased risk of a fatal or severe course of COVID-19 in subjects taking statins (HR 0.70, 95% CI 0.53–0.94) [22]. Interestingly, there is an ongoing randomized trial, Ruxo-Sim-20, assessing ruxolitinib (JAK1 and JAK2 kinase inhibitor) administered with simvastatin on viral entry and decrease in inflammation in subjects with COVID-19 (NCT04348695).

Also, glucagon-like-1 receptor agonists (GLP-1RAs) may play an important role in obese patients with COVID-19. These drugs have been initially used in diabetes treatment but now are also used in the management of obesity itself. GLP-1RAs exhibit anti-inflammatory properties, exert pulmonary protective effects, and have a beneficial influence on, gut microbiome [23]. However, in some animal studies, GLP-1RAs increased ACE-2 levels promoting SARS-CoV-2 infection [24]. Therefore, further well-conducted clinical studies are still in need.

### When one pandemic propels the other

Unfortunately, we observed negative consequences with lockdown and home isolation. De Luis et al. showed an increase in self-reported body weight in obese subjects. It was mainly associated with eating snacks (subgroup eating snacks: 2.60 ± 0.36 vs. subgroup not eating snacks: 1.30 ± 0.17 kg, p < 0.01) in only a 7-week observation [25]. Nowadays, during the COVID-19 pandemic, the rapid upsurge in consumption of high-processed food and decreased energy expenditure in pretty all countries regardless of the income will probably rev up the incidence of overweight, obesity, and other non-communicable diseases in the foreseeable future.

## Conclusions

The cumulative incidence of overweight/obese subjects and the elderly is a crucial problem worldwide. Subjects with overweight and obesity outface a higher risk of detrimental COVID-19 complications, like hospitalization, intensive care management, and death. This is especially true for subjects with morbid obesity. Also, observational studies suggest that in the case of COVID-19, no favorable “obesity paradox” is observed. Inflammatory state, a hallmark of aging and obesity, may play a crucial role in promoting an unfavorable COVID-19 course. Lifestyle, glycemic control, and regulation of the renin-angiotensin-aldosterone pathway have important implications for preventing and managing subjects with COVID-19. And it is crucial to use cardioprotective drugs such as ACE inhibitors/ARB and statins. Nevertheless, there is the need to conduct prospective studies and registries better to evaluate the issue of obesity in COVID-19 patients.

## Data Availability

Not applicable.

## Abbreviations

ACE2: Angiotensin-converting enzyme 2
AT-1: Angiotensin II receptor type 1
CIRCS: Coronavirus disease-related cardiometabolic syndrome
COVID-19: Coronavirus Disease 2019
HR: Hazard ratio
OR: Odds ratio
SARS-CoV-2: Severe acute respiratory syndrome coronavirus 2
